# TNFα induced by DNA-sensing in macrophage compromises retinal pigment epithelial (RPE) barrier function

**DOI:** 10.1038/s41598-023-41610-7

**Published:** 2023-09-02

**Authors:** Michael Twarog, Joshua Schustak, YongYao Xu, Matthew Coble, Katie Dolan, Robert Esterberg, Qian Huang, Magali Saint-Geniez, Yi Bao

**Affiliations:** Department of Ophthalmology, Novartis Institutes for BioMedical Research, 22 Windsor Street, Cambridge, MA USA

**Keywords:** Cell biology, Neuroimmunology

## Abstract

Increasing evidence suggests that chronic inflammation plays an important role in the pathogenesis of age-related macular degeneration (AMD); however, the precise pathogenic stressors and sensors, and their impact on disease progression remain unclear. Several studies have demonstrated that type I interferon (IFN) response is activated in the retinal pigment epithelium (RPE) of AMD patients. Previously, we demonstrated that human RPE cells can initiate RNA-mediated type I IFN responses through RIG-I, yet are unable to directly sense and respond to DNA. In this study, we utilized a co-culture system combining primary human macrophage and iPS-derived RPE to study how each cell type responds to nucleic acids challenges and their effect on RPE barrier function in a homotypic and heterotypic manner. We find that DNA-induced macrophage activation induces an IFN response in the RPE, and compromises RPE barrier function via tight-junction remodeling. Investigation of the secreted cytokines responsible for RPE dysfunction following DNA-induced macrophages activation indicates that neutralization of macrophage-secreted TNFα, but not IFNβ, is sufficient to rescue RPE morphology and barrier function. Our data reveals a novel mechanism of intercellular communication by which DNA induces RPE dysfunction via macrophage-secreted TNFa, highlighting the complexity and potential pathological relevance of RPE and macrophage interactions.

## Introduction

Age-related macular degeneration (AMD) is the most common cause of blindness among the elderly^1^. Increasing evidence indicates chronic inflammatory events (e.g. complement, toll-like receptor—TLR, NFκB, inflammasome, and type I interferon—IFN response) play a central role in the pathogenesis and development of AMD^2,3^, however the molecular nature of the inflammatory mechanisms through which these factors exert their effects remain unclear. The immune status of the chorioretinal interface is maintained by the retinal pigment epithelium (RPE), a critical retinal cell type involved in AMD. Dysfunction and morphological changes of the RPE cells are correlated with AMD^4–6^.

Several reports have demonstrated that the IFN response is elevated in AMD patients, suggested a potential role of nucleic acid (NA) sensing in AMD^7,8^.We previously reported that RPE cells can both produce and respond to type I IFN, primarily through the RNA sensor RIG-I^8^. Although able to mount a robust response to RNA, RPE are unable to do the same in response to DNA due to the absence of canonical sensing machinery such as cGAS^8^. Interestingly, RPE degeneration in an Alu-RNA challenge murine model is blunted when cGAS is inactivated, and mitochondrial DNA may be involved in this process to induce DNA sensing^7^. Those studies suggested that DNA-based NA sensing may also participate in the pathological outcome via other cell types.

Although lacking the central component of DNA sensing machinery, cGAS-deficient cells are nonetheless capable of responding to DNA through interactions with nearby cGAS-expressing cells via the second messenger 2′3′-Cyclic GMP-AMP (cGAMP)^9,10^. Our previous re-analysis of published single-cell transcripts from human donors showed that cGAS was enriched in myeloid cells^8,11^, raising the possibility that macrophage and microglia—the predominant myeloid cells within the human retina—also function to survey DNA levels. We hypothesized that myeloid cells directly sense DNA in the eye via cGAS-STING to activate the type I IFN pathway and to secrete IFNβ, in turn promoting RPE dysfunction.

In this study, we applied a human RPE-macrophage co-culture system to demonstrate that RPE can respond to DNA and activate a type I IFN response through macrophages. Although we find that IFNβ neutralization prevents RPE barrier dysfunction in response to RNA in RPE, it is not sufficient to block DNA-induced barrier dysfunction in this co-culture system. Through profiling of secreted cytokines, we identified TNFα as a key factor that is both secreted from macrophage in response to DNA as well as sufficient to cause DNA-induced RPE dysfunction within the co-culture system. In light of the emerging para-inflammatory roles of macrophage and microglia cells in AMD^12–16^, our findings provide a potential mechanism through which activated myeloid cells damage RPE during disease progression.

## Results

### RPE can respond to DNA through macrophage to initiate type I IFN response

**Figure 1:**
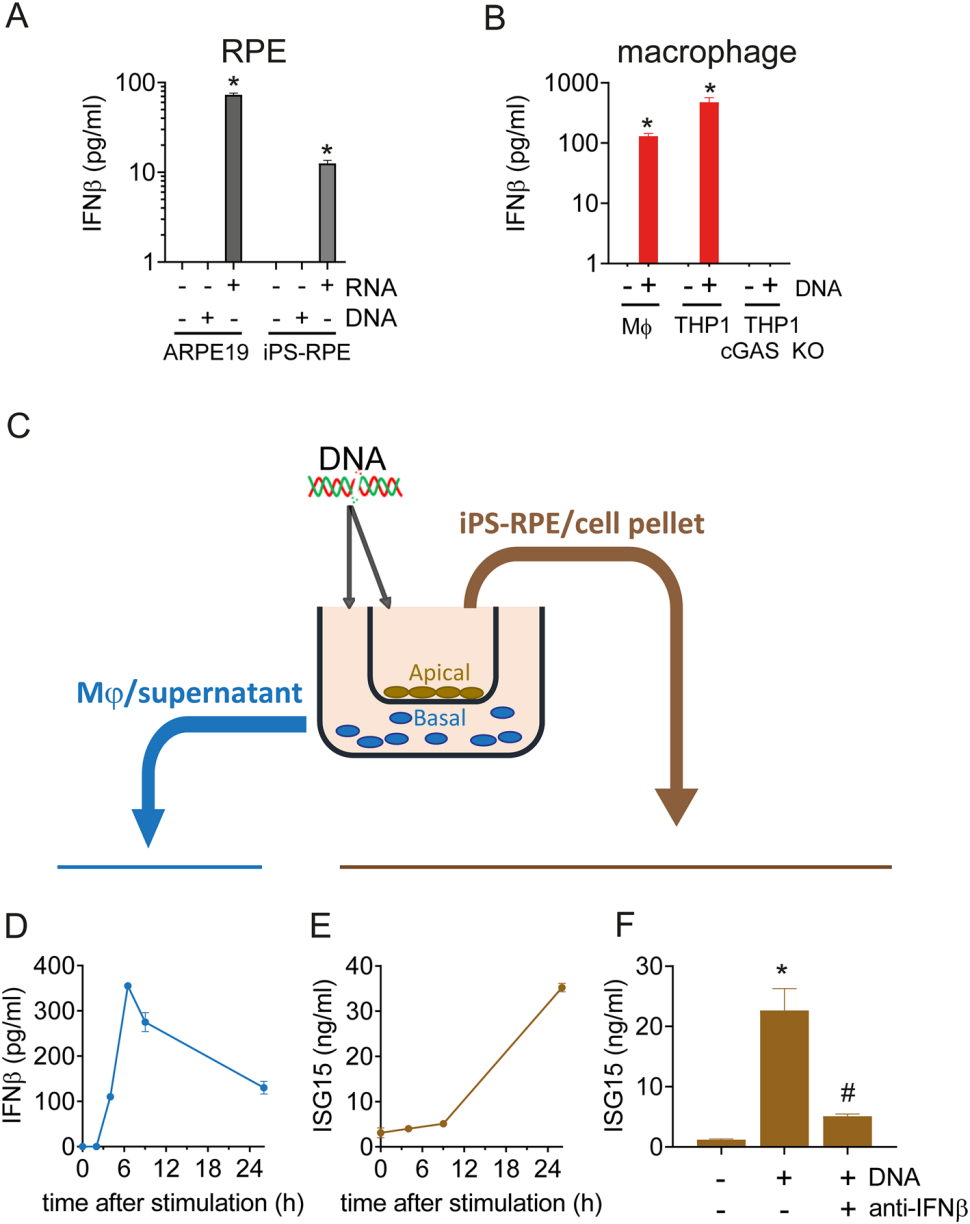
RPE respond to DNA indirectly through macrophages. (**A**) Quantification of IFNβ secretion shows human RPE cells respond to direct RNA (3p-hpRNA, 50 ng/ml) exposure but not DNA (dsDNA-EC, 500 ng/ml). (**B**) IFNβ secretion in human primary macrophages and THP1 cells in response to DNA (dsDNA-EC, 500 ng/ml) is abrogated in cGAS KO THP1 cells. (**C**) Schematic diagram of the macrophages-RPE co-culture system. (**D**) Macrophage-derived IFNβ secretion following DNA exposure (dsDNA-EC, 500 ng/ml) over 24 h period. (**E**) RPE-derived ISG15 secretion in response to DNA (dsDNA-EC, 500 ng/ml) treatment over 24 h period. (**F**) Anti-IFNβ attenuates ISG15 production from RPE in response to DNA exposure in this co-culture system. Each data point represents biological replicates (n = 3), and are reported as mean ± S.D. *p < 0.05 compared to relative vehicle control groups. # *p* < 0.05 compared to DNA stimulated group.

We previously found that RPE respond to RNA directly through RIG-I, but cannot directly respond to DNA through cGAS-STING pathway due to the absence of cGAS expression (see reference and Fig. 1A). The RPE selective response to RNA is thus contrasting with recent mouse studies demonstrating a key role of cGAS in Alu-RNA-mediated RPE degeneration^7^. Since RPE participate in a self-propagating feedback loop in response to IFNβ^8^, we hypothesized that RPE indirectly sense DNA through cell–cell interactions.

To identify the cells responsible for mediating DNA sensing within the retina, we re-analyzed published single-nucleus RNA-seq of human donor retinal tissue to search for potential cGAS-expressing cells^8,11^, which point to macrophages as a potential cell type of interest. Using monoculture system, we first confirmed that macrophages indeed sense DNA through cGAS and generate IFNβ (Fig. 1B). Multiple sources and types of dsDNA, including commercially available synthetic DNA, bacterial DNA, and eukaryotic DNA isolated from THP1 cells, were tested on THP1 cells, a monocyte cell-line model for macrophage function, and primary human macrophages. All were able to activate an IFN response in these macrophages, albeit at different levels (Supplementary Fig. 1). We selected dsDNA-EC, a bacterial dsDNA which elicited the strongest response in THP-1, as the cGAS agonist for this study. Next, we established a co-culture system to better understand myeloid-RPE interactions in the context of NA sensing (Fig. 1C). In this system, we observed that DNA stimulates a robust IFN production in macrophages, peaking 4–6 h following DNA exposure (Fig. 1D). ISG15, an early canonical interferon-stimulated gene (ISG), was used to evaluate IFN response in RPE. Interestingly, ISG15 was detected in RPE after DNA treatment in this co-culture system, much later than the IFNβ peak in macrophages (Fig. 1E). Importantly, this downstream signaling of type I IFN response was attenuated by anti-IFNβ neutralizing antibody (Fig. 1F), confirming that the initiation of these events occurs through DNA-sensing induced IFNβ.

### DNA-sensing-induced response in macrophages impairs RPE barrier function

**Figure 2:**
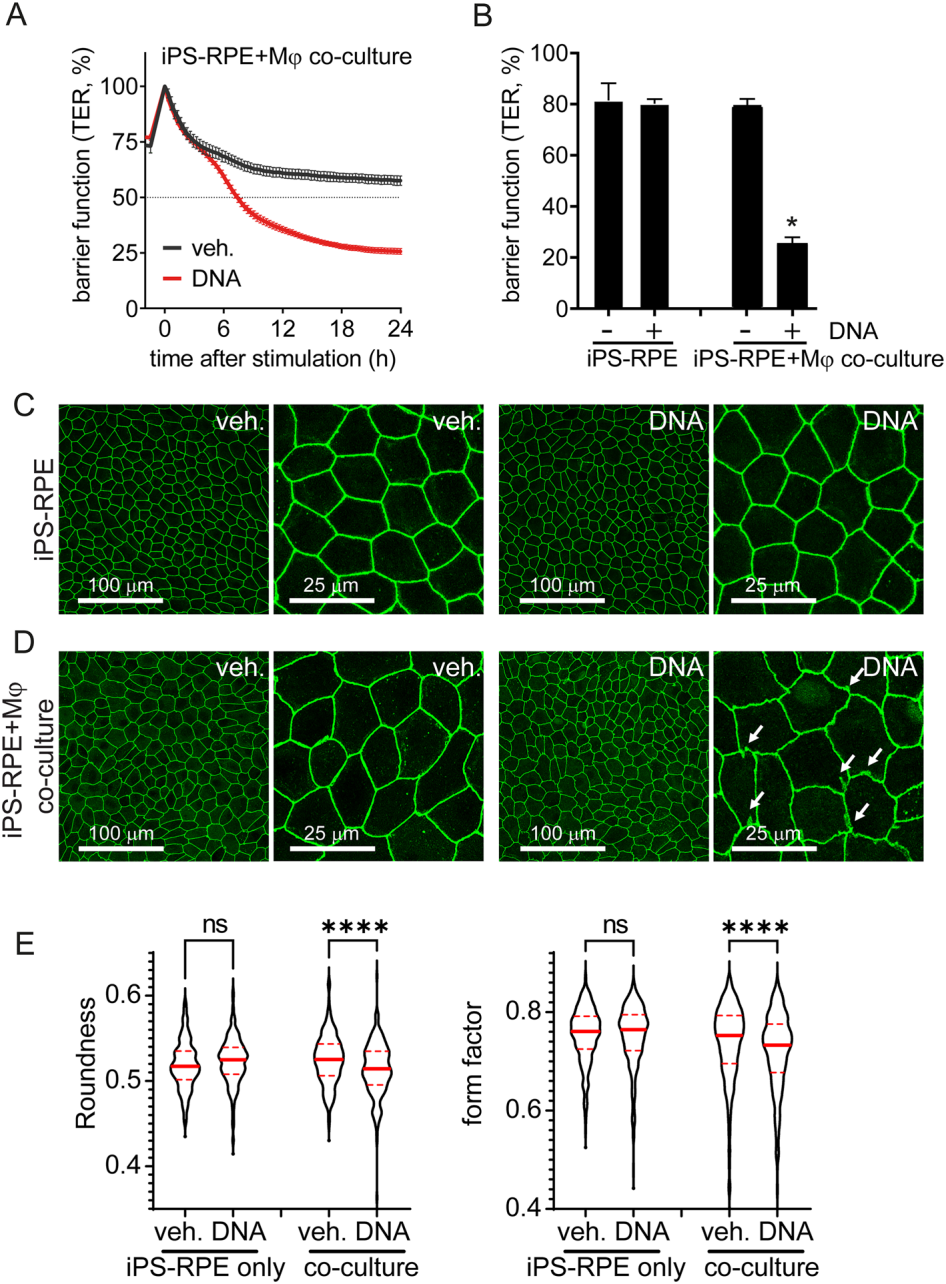
Indirect impairment of RPE barrier function via DNA sensing response in macrophages. (**A**,**B**) RPE barrier function was assessed by transepithelial resistance (TER) measurement. TER was reduced in response to DNA (500 ng/ml) in human iPS-RPE-macrophage co-culture system. Each data point represents biological replicates (n = 3), and reported as mean ± S.D..* *p* < 0.05 compared to relative vehicle control groups. (**C**–**E**) RPE morphology and tight-junctions were evaluated by ZO-1 staining. DNA treatment of iPS-RPE alone has no effect on the cells’ morphology (**C**), but DNA-sensing-induced macrophage activation impairs RPE cells’ shape and ZO-1 membranous localization (zigzag and intracellular aggregates) in the co-culture system (**D**). Representative pictures were selected from 3 independent experiments. (E) RPE roundness and form factor were analyzed from at least 3 images for each group. Values of individual cell were used to generate a violin plot with median and quartile ranges. NS, not significant; ****, *p* < 0.0001.

To better understand the biological consequence of NA sensing on RPE cells, we set out to evaluate RPE barrier integrity, a key function of RPE cells^17,18^. We use transepithelial resistance (TER) of polarized iPS-RPE cells cultured on a permeable transmembrane matrix to characterize the RPE barrier function. Previously we showed that RNA, but not DNA stimulation, could disrupt barrier function of human RPE cells^8^. As shown in Fig. 2A,B, application of our macrophage co-culture system shows that macrophages respond to DNA which consequently impede RPE barrier function.

RPE barrier function is enabled by apical tight-junctions, thus we investigated junctional integrity by visualization of the marker Zonula occludens-1 (ZO-1). Under unchallenged conditions, RPE cells form a characteristic cobblestone monolayer with defined cell borders (Fig. 2C). When DNA was applied directly to RPE cells, no gross changes in RPE morphology was detected (Fig. 2C,E), as expected in cell lacking cGAS expression^8^. Yet, when macrophages and RPE co-cultures were exposed to DNA, RPE morphology appeared strongly compromised (Fig. 2D,E), consistent with our TER observations. Measurement of morphological parameters revealed a significant decrease in cellular roundness and form factor, two widely used RPE morphometric parameters^19–21^, following DNA treatment. Interestingly, these changes were associated with re-organization of RPE tight-functions with jagged and zigzag redistribution of ZO-1 (Fig. 2D,E). These findings suggest that RPE barrier and morphometric changes are caused by factors secreted from macrophages in response to DNA challenge.

### Anti-IFNβ does not prevent barrier function loss or restore morphology in RPE cells in a macrophage-RPE co-culture model treated with DNA

**Figure 3:**
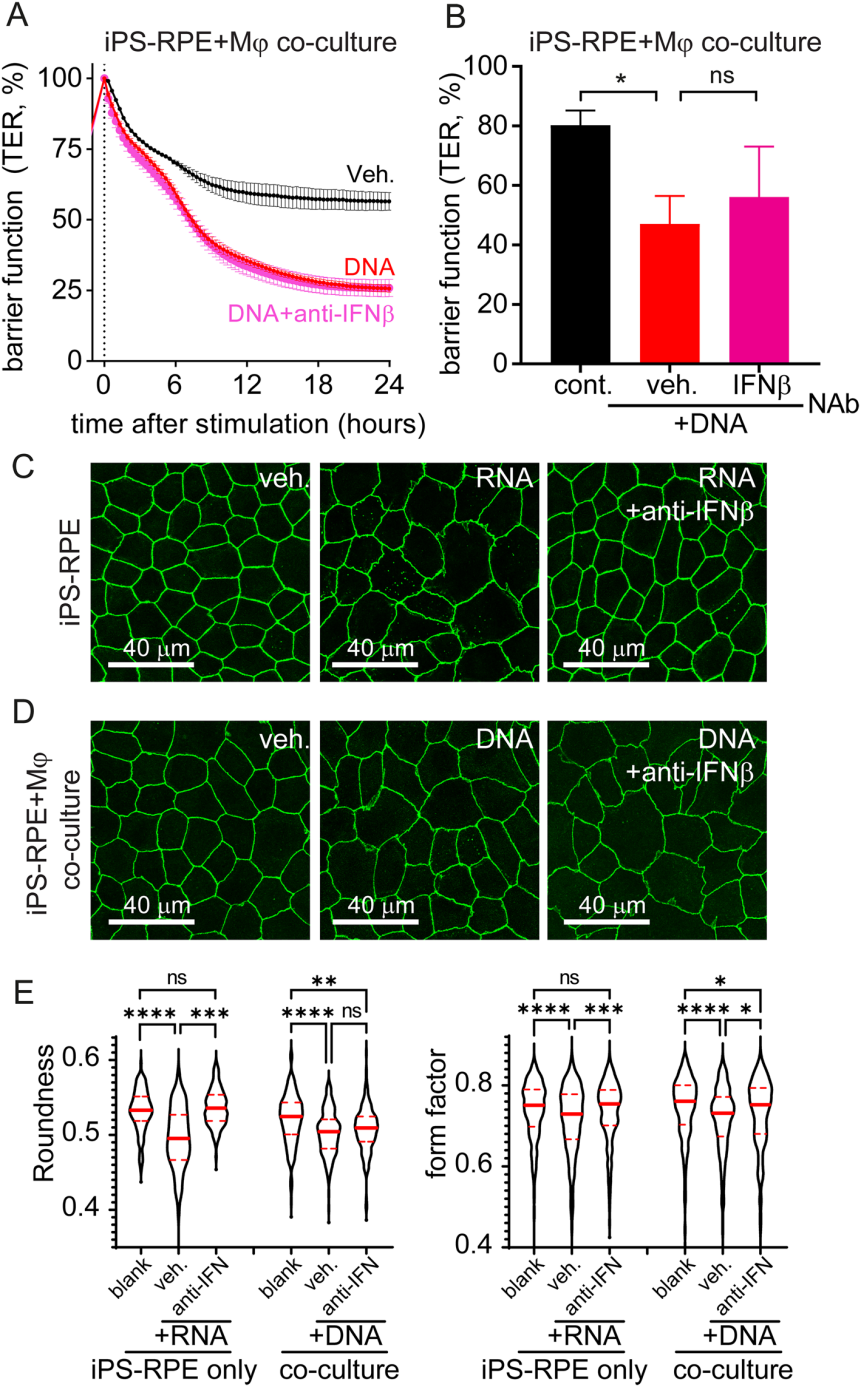
IFNβ neutralization does not prevent DNA-induced RPE barrier disruption in co-culture system. (**A**,**B**) RPE barrier function was assayed by transepithelial resistance (TER) measurement. Anti-IFNβ neutralizing antibody (NAb, 5 μg/ml) does not prevent DNA-induced (500 ng/ml) TER loss in the iPS-RPE-macrophage co-culture system. An example of time curve from one independent study is shown in panel (**A**), and results from 3 independent studies at 18 h are summarized in panel (**B**). Each data point represents biological replicates (n = 3), and reported as mean ± S.D. *p < 0.05 compared to relative vehicle control groups. (C-E) RPE shape and tight-junction morphology were evaluated by ZO-1 staining. RNA exposure induced iPS-RPE amorphic changes and tight-junctions remodeling that are rescued by IFNβ neutralization (**C**). Anti-IFNβ did not prevent RPE changes following DNA challenge and activation of macrophages in co-culture conditions (**D**). Representative pictures from 3 independent experiments. (**E**) Violin plot of RPE roundness and form factor (data from at least 3 images per condition). NS, not significant; *p < 0.05; **p < 0.01; ***p < 0.001; ****p < 0.0001.

Next, we characterized the mechanism by which macrophages impact RPE biology via DNA sensing. As we have previously reported, anti-IFNβ neutralizing antibody prevents RNA-induced barrier function loss measured by TER in RPE cells, suggesting a direct involvement of the IFN pathway^8^. Thus, we assessed whether IFNβ produced by macrophage could also be responsible for compromising RPE barrier function in the co-culture system. Surprisingly, although anti-IFNβ antibody successfully reduced DNA induced IFN response in this co-culture system when measured by ISG15 (Fig. 1F), IFNβ neutralization did not prevent DNA-mediated RPE TER loss in the same conditions (Fig. 3A,B). Similarly, investigation of RPE morphology with ZO-1 staining confirmed the ability of IFNβ neutralizing antibody to prevent RNA-induced amorphic changes (Fig. 3C,E), while having little to no effect when applied to the co-culture system (Fig. 3D,E), further supporting our suspicion that other factors in addition to IFNβ produced from macrophages are involved in this process.

### TNFα, induced by DNA sensing in macrophage, is the major factor affecting RPE barrier function

**Figure 4:**
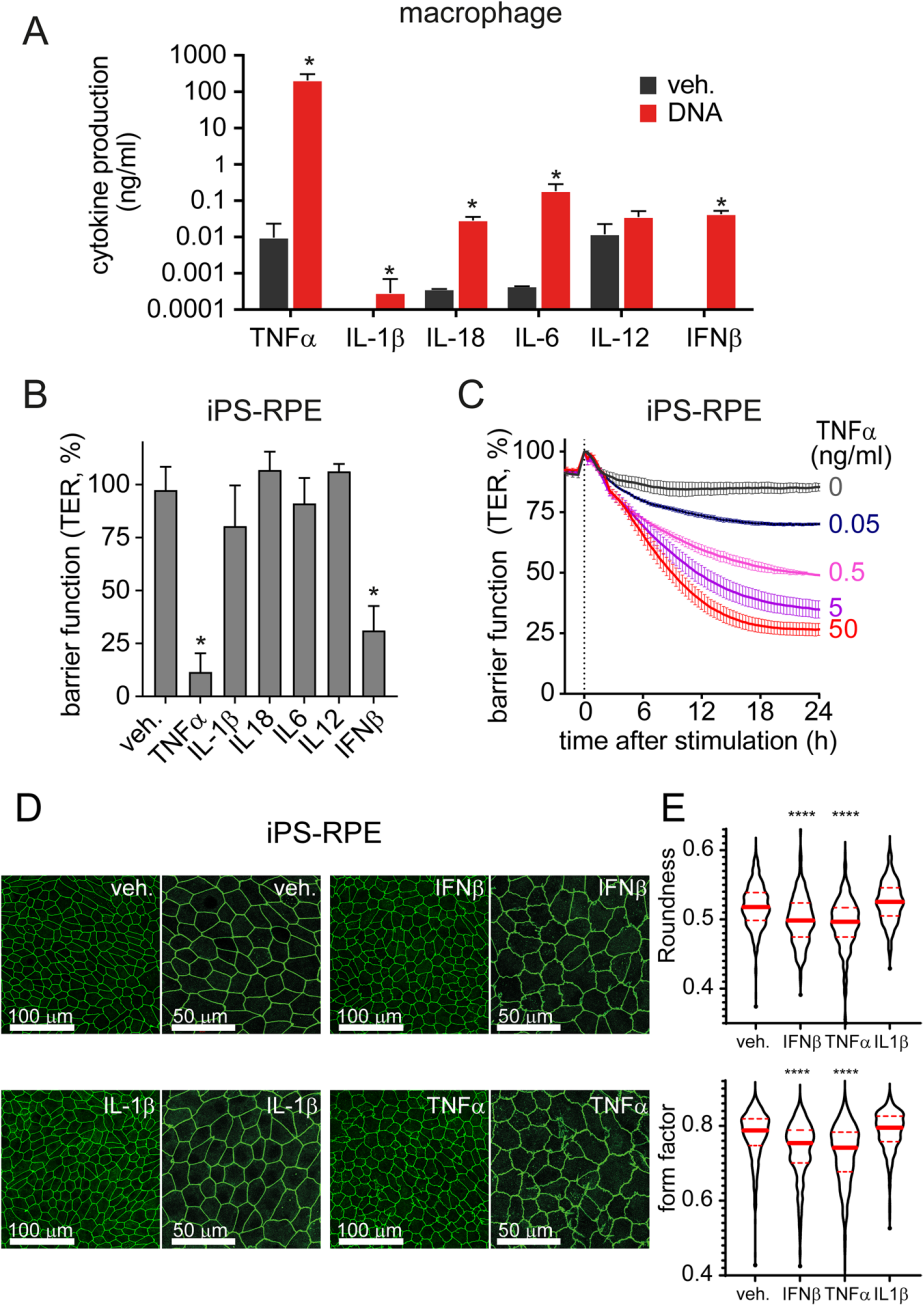
IFNβ and TNFα, secreted from macrophages in response to DNA, cause RPE barrier function loss. (**A**) Quantification of pro-inflammatory cytokines secreted by macrophages in response to DNA (500 ng/ml) at 24 h. (**B**) Evaluation of the effect of macrophage-derived cytokines on RPE TER shows that both IFNβ and TNFα alter RPE barrier function. Different cytokines (100 ng/ml) were applied on iPS-RPE, and RPE barrier function was measured by transepithelial resistance (TER) assay. Data was shown the percentage changes of TER at 18 h. Each data point represents biological replicates (n = 3), and indicated as mean ± S.D..*p < 0.05 compared to relative vehicle control groups. (**C**) Dose–response of TNFα was evaluated on RPE barrier function. (**D**,**E**) RPE morphology and tight junctions were evaluated by ZO-1 staining. IFNβ and TNFα (both at 100 ng/ml) induced tight junctions remodeling in iPS-RPE at 24 h. Representative pictures were shown from 3 independent experiments. (**E**) Violin plot of RPE roundness and form factor (data from at least 3 images per condition). ****p < 0.0001 compare to vehicle group (veh).

In order to identify the mediators of macrophage-induced RPE barrier disruption, we measured a panel of cytokines to determine which ones are secreted by macrophages following stimulation with DNA. We observed different pro-inflammatory cytokines released into the medium in response to DNA stimulation. Specifically, IL-18, IL-6 and IFNβ were all detected with levels measured in pg ~ ng/ml. Interestingly, TNFα was the highest detected cytokine (100 ng–1 µg/ml) after stimulation with DNA in macrophages (Fig. 4A).

To determine the functional consequence of macrophage-derived cytokines on RPE barrier function, we added each of these pro-inflammatory cytokines (100 ng/ml) onto RPE. A partial reduction in barrier function was achieved with IFNβ at 18 h (Fig. 4B), as we have shown previously^8^. Surprisingly, TNFα was the only other cytokine assayed to exert a dose–response effect on TER, while IL-1β, IL-6, IL-12, and IL-18 had no detectable effect (Fig. 4B,C). Evidence of altered tight junction organization were replicated through ZO-1 staining. Only IFNβ and TNFα induced irregular RPE morphology (Fig. 4D,E), suggesting that TNFα may play a key role in regulating RPE barrier function, which align with previous publications^22,23^.

**Figure 5:**
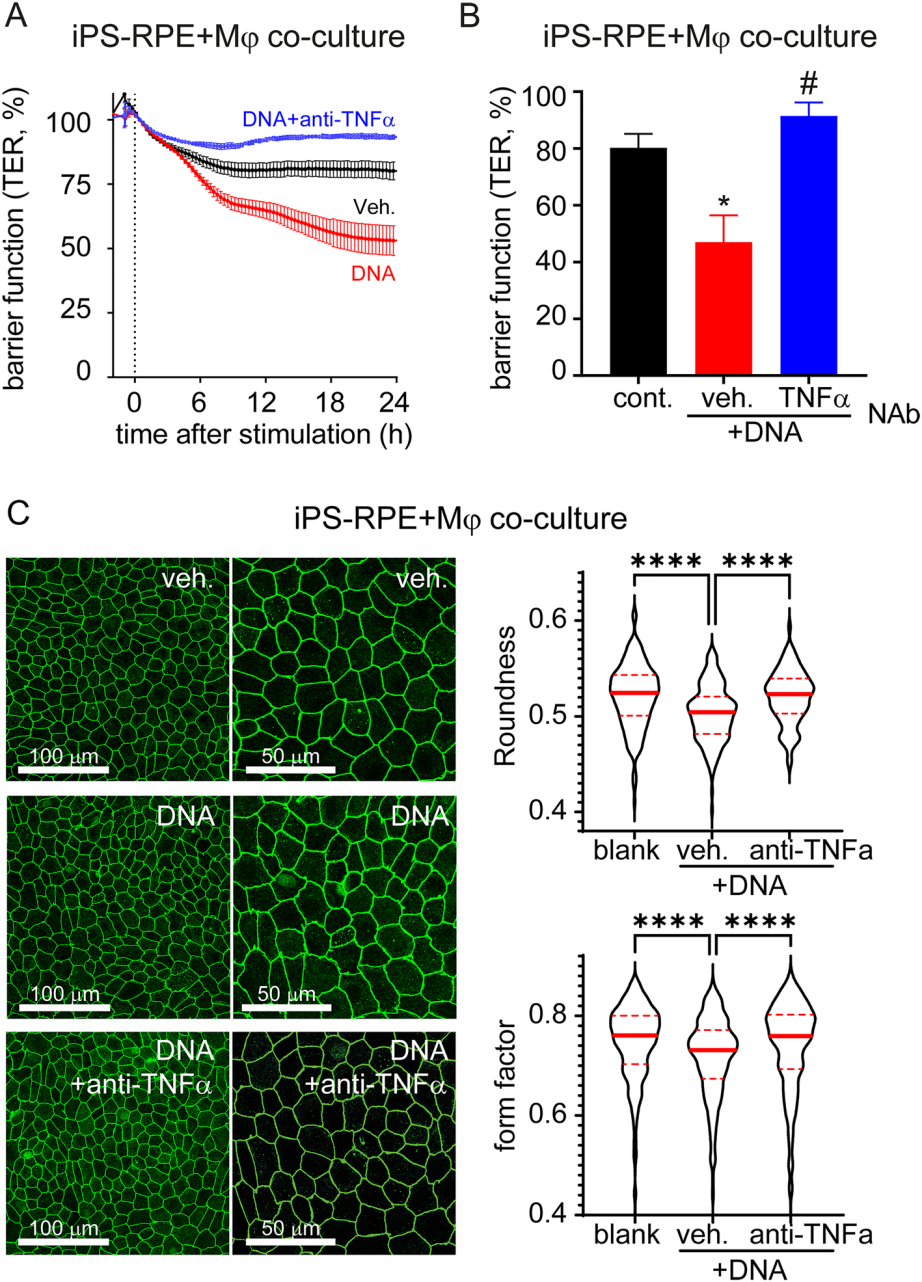
TNFα neutralization prevents DNA-sensing-induced RPE barrier function defects in an iPS-RPE-macrophage co-culture system. (**A**,**B**) RPE barrier function was measured by transepithelial resistance (TER) assay. Anti-TNFα (at 5 µg/ml) prevents DNA-induced (500 ng/ml) TER loss in iPS-RPE-macrophage co-culture system. An example of time curve from one independent study was shown in panel A, and results from 3 independent studies at 24 h were summarized in panel B. Each data point represents biological replicates (n = 3) and reported as mean ± S.D. *p < 0.05 compared to relative vehicle control groups. (**C**) RPE shape and tight-junction morphology were evaluated by ZO-1 staining. Anti-TNFα (at 5 µg/ml) suppresses the iPS-RPE morphometric changes triggered by DNA-sensing and activation of macrophages in the co-culture system (at 24 h). Representative pictures selected from 3 independent experiments. Violin plot of RPE roundness and form factor (data from at least 3 images per condition). ****p < 0.0001.

To further confirm this finding, we blocked TNFα or other cytokines in our macrophage-RPE co-culture system using neutralizing antibodies. DNA-sensing induced barrier function defects were measured by both TER and ZO-1 staining (Fig. 5). Anti-IFNβ antibody had little effect on DNA-induced RPE barrier function loss in co-culture system (Fig. 3D,E). In contrast, anti-TNFα neutralizing antibody completely rescued DNA-induced TER loss (Fig. 5A,B) and morphology changes (Fig. 5C). These results indicate that TNFα released from DNA-induced macrophage can alter RPE barrier function.

### Cross talk between TNFα and IFNβ pathways

Previous reports indicate that IFNβ and downstream signaling are activated in AMD patients^7,8^. Here we identified TNFα induced by DNA sensing in macrophages as a major pro-inflammatory cytokine impairing RPE barrier function in vitro, so we sought to investigate molecular signals of TNFα activation from fresh human AMD donor samples. TNFα signature genes, including TNFAIP3, EGR1 and SERPINE3, were quantified in macular retina and RPE-enriched samples from patients with different grades of AMD. Although no statistical significance could be measured between AMD grades, a general trend toward induction was observed as various AMD grades above the non-AMD controls (AMD1) (Supplementary Fig. 2), suggestive of a general correlation between AMD and TNFα activity.

**Figure 6:**
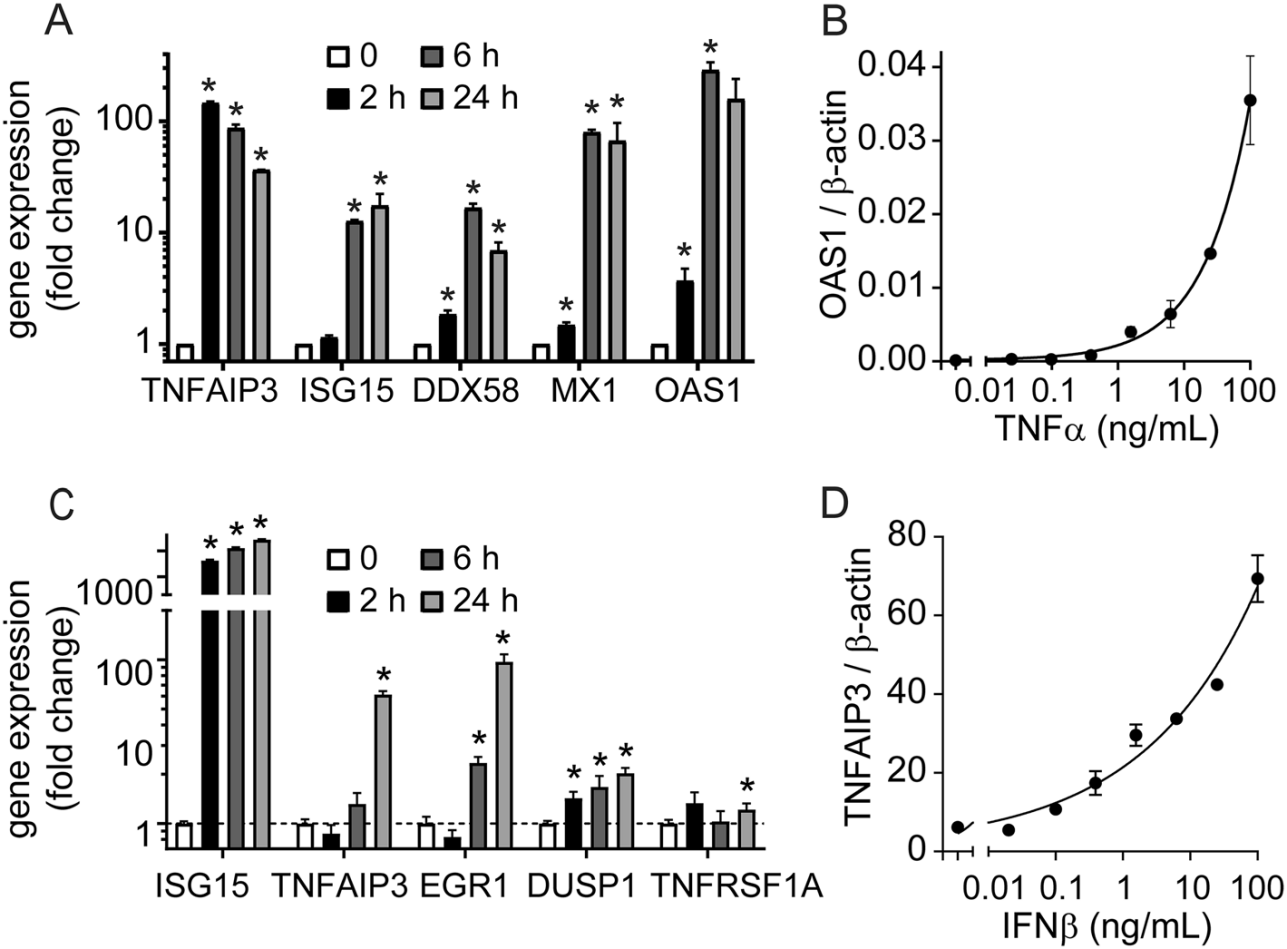
Crosstalk between TNFα and IFNβ pathways in RPE. (A-B) TNFα induces IFN response in iPS-RPE cells in a time- (**A**) and dose- (**B**) dependent manner. (**C**,**D**) IFNβ induces TNF response in iPS-RPE cells in a time- (**C**) and dose- (**D**) dependent manner. ISG15 was used as a marker for IFNβ pathway activation. Each data point represents biological replicates (n = 3), and results are reported as mean ± S.D. *p < 0.05.

Since both TNFα and IFNβ signatures appear to be increased in AMD patients, we sought to evaluate a potential relationship between the two pathways. It has been reported that TNFα can activate a type I IFN response and induce ISGs through IRF1 in different macrophages or fibroblast-like synoviocytes^24–26^, so we evaluated whether a similar mechanism was applicable to RPE. iPS-RPE cells were treated with TNFα at indicated doses and times. TNFAIP3 (TNF Alpha Induced Protein 3 or A20), was used as a marker of TNFα activity^27,28^. The TNFAIP3 expression was rapidly induced after 2 h of treatment with TNFα (Fig. 6A). Next, we measured several ISGs, including ISG15, DDX58, MX1 and OAS1 by qPCR, to indicate activation of IFN pathway. The results showed that following TNFα pathway activation (2 h post-treatment), the IFN pathway was activated, though delayed, as most biomarkers peaked after 6 h in RPE cells (Fig. 6A,B).

Conversely, we investigated the effect of IFNβ on TNF pathway. We treated iPS-RPE cells with IFNβ at indicated dose and time, and quantified IFN and TNF signature genes. ISG15 was used as a positive control for IFN response and TNFAIP3, EGR1, DUSP1, and TNFRSF1A were used as molecular biomarkers of TNF pathway activation. As expected, ISG15 was strongly and rapidly induced in RPE following IFNβ exposure (Fig. 6C). Interestingly, TNF pathway was also activated in RPE cells but in a delayed manner with most TNF associated genes induced at 24 h (Fig. 6C,D).

## Discussion

In eukaryotic cells, levels of NA including DNA and RNA, are closely monitored and actively maintained by intrinsic regulators. As powerful mediators of host defense machinery, such regulators must also sense and respond to endogenous levels of NA present within the cell. Aberrant accumulation of self-derived NA molecules can also trigger maladaptive host responses leading to sterile inflammation^29^. In mammalian cells, recognition of NA involves multiple sensing machineries. Intracellular RNA sensing occurs mainly through RIG-I-like receptors (RLRs), a group of RNA-specific pattern recognition receptors (PRRs). Intracellular DNA sensing, however, occurs mainly through the cGAS/STING axis, which detect cytoplasmic double-stranded DNA (dsDNA)^30,31^. Once activated, NA sensors trigger multiple signaling cascades, and produce type I IFNs and pro-inflammatory cytokines via interferon regulatory factor (IRF) and NFκB transcription factors^32,33^. Our previous study showed that NA sensing within RPE cells is governed primarily by the RNA sensor RIG-I, and not through cGAS as previously reported^7,8^. Interestingly, an investigation of a cGAS KO mouse model indicated cGAS genomic deletion could protect RPE from degeneration caused by Alu-RNA mediated mitochondrial DNA (mtDNA) release, suggesting a role of DNA sensing in AMD as well^7^. These seemingly contradictory findings suggest that other retinal cell populations expressing cGAS might directly respond to accumulating immunogenic NA and propagate damage-inducing pro-inflammatory signals.

In support of this hypothesis, DNA-sensing cells can elicit a paracrine response and indirectly activate neighboring cells via IFN secretion or the second messenger cGAMP generated by cGAS^9,10^. In ocular tissues, the major cell types expressing cGAS are of the myeloid lineage^8,11^. Phagocytes, especially monocyte/macrophage and microglia, surveil the choroidal and subretinal space and thus are essential to maintaining retinal homeostasis. Due to their close proximity with RPE, phagocytes may also cause or exacerbate retinal degeneration in ocular diseases such as retinitis pigmentosa and age-related macular degeneration^12–16^. This complexity of RPE-macrophage communication leads to challenges in interpreting transcriptomic analyses. Using bulk RNA collected from AMD patients, we investigated the inflammatory profile changes during disease progression of key inflammatory markers by qPCR. Key markers of TNFα, TNFAIP3, EGR1, and SERPINE3 showed a trend towards induction with AMD stage. However, the contribution of individual cell populations to this response remained elusive (Supplementary Fig. 2). Investigating cell–cell interactions using a human macrophage and RPE cells co-culture model enabled the identification of an indirect role for DNA sensing in RPE through proximal macrophages. RPE can only respond to DNA and mount a type I IFN response when co-cultured with macrophages, indicating an indirect role for the cGAS/STING axis in this process.

**Figure 7:**
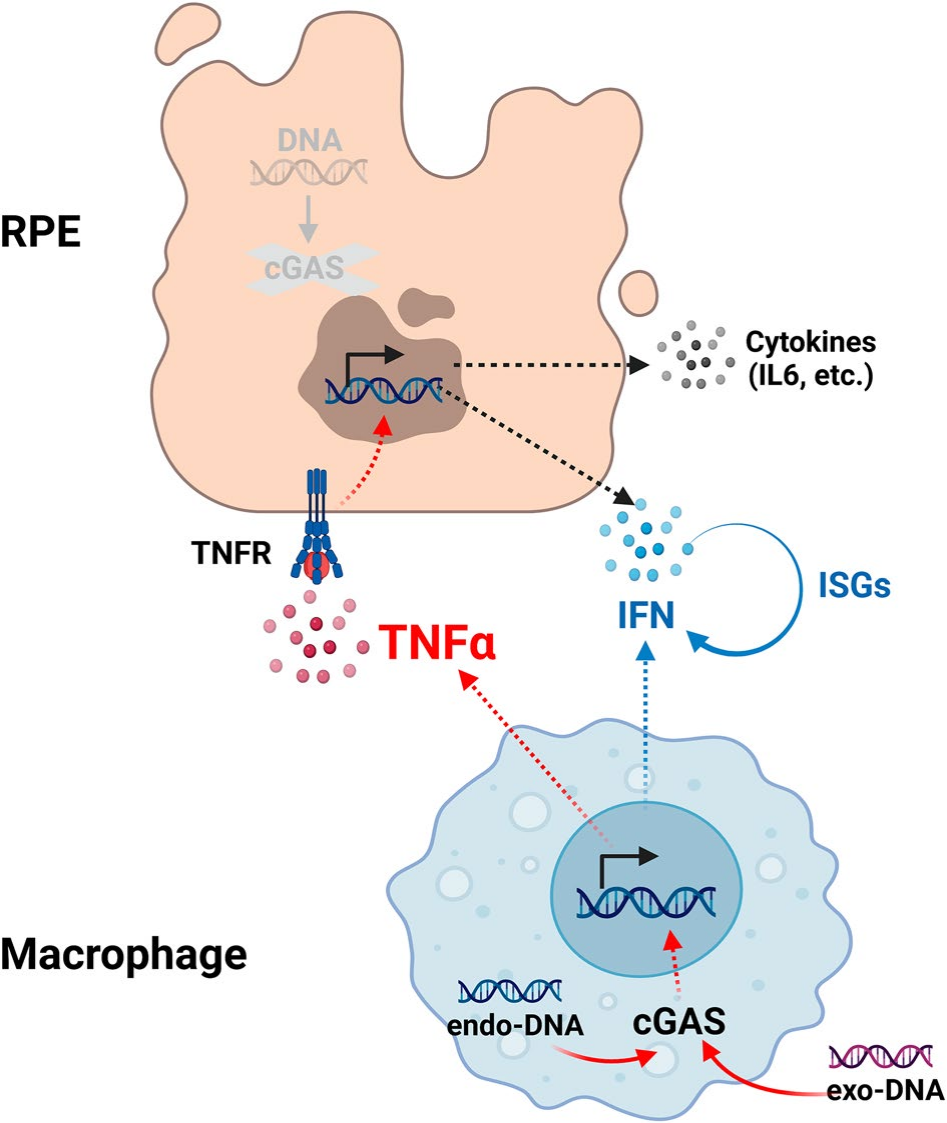
Schematic mechanism of RPE response to DNA-dependent macrophages activation. RPE cells lack the DNA sensor cGAS and are unable to directly respond to immunogenic DNA. Instead, RPE cells can respond to DNA indirectly via macrophage. cGAS-expressing macrophages respond to exogenous (exo-) or endogenous (endo-) DNA sources, triggering the secretion of inflammatory cytokines, including TNFα. TNF receptors (TNFR) on RPE can detect secreted TNFα, resulting in inflammation, disruption of RPE barrier function, and RPE de-differentiation. Additionally, the production of IFN and ISGs from both RPE and macrophage, secondary to indirect and direct response to DNA, further promotes inflammation. Figure created with BioRender.com.

Adding to our previous demonstration of a direct disruptive effect for RNA on RPE barrier function^8^, we show that DNA indirectly induces RPE barrier defects and tight-junctions disorganization through macrophage activation. Importantly, we found that while IFNβ neutralization is able to efficiently inhibit DNA-induced IFN signaling in macrophages (Fig. 1F), it had little effect on RPE barrier dysfunction in this co-culture system (Fig. 3). This argues that other factors secreted from macrophages play a more significant role in inducing RPE dysfunction. Using this human macrophage-RPE co-culture system, several proinflammatory cytokines were evaluated in our study, and TNFα was identified as the key regulator as it is both secreted from macrophage in response to DNA and a potent mediator of RPE barrier dysfunction. A schematic diagram (Fig. 7) was included to provide a concise summary of the proposed mechanism of action.

Beside secreted cytokines, other signaling factors may also be involved in the indirect macrophage to RPE NA sensing process we unveiled. cGAMP, a second messenger synthesized by cGAS in response to DNA may also activate a type I IFN response in RPE. Indeed, it was reported that cGAS-deficient cells can respond to DNA indirectly with neighboring cGAS-expressing cells through intercellular cGAMP transfer via connexin channels^9^. In addition to trafficking through connexin channels^10^, cGAMP can also signal between cells through micro-vesicles or exosomes^34^. Interestingly, exosome markers were found in drusen from AMD donors^35^. As transfer organelles for various protein and molecules, including second messengers, DNA, mRNA, and microRNAs, exosomes may represent a critical mode of intercellular communication in the outer-retina^36–39^. Further investigation on the interplay between DNA sensing and exosomal cargo loading and secretion by macrophages may reveal novel mechanisms for RNA sensing induction in RPE.

As mentioned, the origin and identity of immunogenic NA in AMD remains highly elusive. Several theories have been previously proposed, including released mtDNA. Mitochondrial dysfunction is a hallmark of AMD progression^40^, and the release of mtDNA has been shown to illicit an immune response in several cell types^41^. During stress conditions, such as oxidative stress, mtDNA can be released from mitochondria into the cytoplasm through megapores such as BCL2 or VDAC^41^. BCL2 has been implicated in RPE apoptosis in response to oxidative stress^42^, and provides a potential mechanism for RPE-derived extracellular DNA. Alternatively, the source of extracellular DNA may not be from the RPE, but rather from circulating DNA, which has been shown to increase with age^43^. Further investigation is necessary to deconvolve the origin of extracellular DNA in AMD.

Our study was focused on only macrophages and RPE interaction and thus may lack important contributions from other ocular cell types, which could also participate in NA sensing and response in this complex cellular ecosytem. The DNA sensor cGAS is expressed in most myeloid cells, including both macrophage and microglia, which have been reported to perhaps play an important role in retinal physiology and pathology^13,16^. In addition, the RPE interacts closely with choroid, so cells from circulation and endothelial cells may also have an impact on this NA sensing process. In future studies, these other cell types can be evaluated in a co-culture system to elucidate their contribution to RPE function. Additionally, direct cell–cell contact between RPE and macrophages was not modeled in this study. While the ratio of RPE to macrophages in human eyes is predicted to be about 10:1^15,44^, we used a ratio of 2:1 (600 k RPE and 300 k macrophage per well) to simulate a local event of macrophage accumulation around damaged RPE cells during disease conditions. This ratio was determined by volumetric parameters in the transwell system that impacted the molarity of macrophage-produced TNFα in the medium (Supplementary Fig. 1D and Fig. 4C). While we acknowledge the limitations of our in vitro system compared to the complexity of the human eye, developing more clinically relevant co-culture systems allowing the study of direct cell–cell contact at physiologic ratios, and evaluating this mechanism using in vivo models would greatly improve our understanding of the interaction between these two cell types^45^.

TNFα is a proinflammatory cytokine that coordinates tissue homeostasis by regulating cytokine production, cell survival, and cell death. It promotes inflammatory responses, which causes many of the clinical problems associated with autoimmune disorders such as rheumatoid arthritis, inflammatory bowel disease, psoriasis, refractory asthma, etc. An association was reported between rheumatoid arthritis diagnosis and increased AMD diagnosis^46^, and another report showed increased plasma TNFα in complement factor H (CFH) AMD-risk variant carriers^47^, pointing out a possible role for TNFα in AMD. TNFα has been shown in vitro to acutely impair RPE barrier function and induce dedifferentiation after chronic exposure^22,23^. In addition, it was reported that monocyte-derived macrophages from wet AMD patients have a great amount of TNFα^48^, which aligns with our hypothesis that TNFα generated from macrophages may promote RPE damage and AMD progression to late stages. Here, we show that TNFα is a potent inducer of IFN while IFN itself is not as efficient at inducing TNFα (Fig. 6). Our analysis of human samples showed an AMD grade-dependent increase trend, albeit insignificant, in TNFα (Supplementary Fig. 2). This may reflect the role of macrophage-derived TNFα as an initiator of AMD, setting off an IFN signaling loop that drives disease progression.

Overall, our data support a role for TNFα in inducing RPE damage and suggest that inhibiting TNFα directly or indirectly via targeting NA sensing may provide therapeutic benefit for patients with retinal diseases associated with inflammation-driven RPE degeneration.

## Material and methods

### Human samples

Postmortem human eyes, including patients with different grades of AMD determined ex vivo using the Minnesota Grading System, were procured from the Lions Eye Institute for Transplant & Research (LEITR; Tampa FL) with consent of donors or donors’ next of kin and in accordance with the law of US and Florida, the Declaration of Helsinki, FDA regulations, and Novartis guidelines of using human tissues in research. All donor tissues were collected by Lions Eye Institute and preserved by snap freezing within 6 h postmortem (n = 9–13 for retina samples and n = 8–26 for RPE enriched samples for each grade of AMD). Macular retina and scrapped RPE (RPE enriched) tissues were freshly dissected for transcript analysis by qPCR. Briefly, eligible globes were cut circumferentially at the pars plana and the anterior segment and vitreous removed. Under a dissecting microscope, a 6 mm tissue biopsy punch was carefully applied on retina by aligning the center of the punch to fovea and was gently pressed to only cut through retina and choroid avoiding the sclera. Next, the macular retina was removed from the punch, peeled from the RPE and processed for RNA extraction. The RPE cells inside of macular punch mark were then carefully scraped off from the choroid tissue using a 1 mL pipet tip and floated in PBS. The macular RPE containing PBS solution was collected into a centrifuge tube and the RPE-enriched macular cells pellet harvested by centrifugation, and processed for RNA extraction.

Human blood was obtained from Medcor Cambridge Research Donor Program using the Western Institutional Review Board (WIRB) Protocol #2015286 and WIRB-approved patient informed consent. The WIRB protocol complies with all federal, state, and local laws pertaining to human research. We acquired blood samples from healthy individuals aged 18–65 under patient informed consent, in accordance with the Declaration of Helsinki ethical principles for medical research involving human subjects. The study protocols were approved by the Novartis Research Center ethical committee.

### Preparation and culture of primary macrophage

Human blood was collected into EDTA-coated tubes. Peripheral blood mononuclear cells (PBMCs) were isolated using Ficoll-Paque Plus density centrifugation (500 g for 30 min at room temperature without brake). Monocytes were purified using the Monocyte isolation kit II from Miltenyi Biotec (Charlestown, MA). Non-monocytes were magnetically labeled using a cocktail of biotin-conjugated antibodies as well as anti-Biotin MicroBeads. Monocytes were enriched by depletion of the magnetically labeled cells through a MACS Separator (Miltenyi Biotec). All macrophage cultures were carried out in macrophage media (Promocell, Heidelberg Germany) in the presence of human recombinant GM-CSF or M-CSF (100 ng/ml unless indicated otherwise) for at least 7 days.

### Cell lines and iPS-derived cell cultures

The human RPE cell line, ARPE-19, was purchased from ATCC (Manassas, VA; CRL-2302). ARPE-19 cells were expanded in DMEM/F12 (Gibco Life Technologies, Carlsbad, CA) supplemented with 10% heat-inactivated fetal calf serum (FBS, Sigma-Aldrich, St. Louis, MO) and 1% penicillin/streptomycin (Gibco BRL, Grand Island, NY) at 37 °C and 5% CO_2_. Cells were matured in serum free RtEBM medium (Lonza, Basel, Switzerland) at least 3 weeks post high density seeding to form mature monolayers.

iCell-RPE (Donor #01279), an iPS-derived RPE cell line, was purchased from FUJIFILM Cellular Dynamics (Madison, WI). Cells were expanded in RtEBM medium supplemented with 2% FBS at 37 °C and 5% CO_2_. iPS-RPE cells were cultured on tissue culture treated plates, or on Falcon transmembrane inserts for polarization (Corning, Corning, NY; 353,095). Cells were maintained at least 3 weeks post-seeding in serum free RtEBM media to form mature monolayers. Maturation of cells was confirmed by pigmentations, immunofluorescence staining for ZO-1 and BEST1, and transepithelial resistance^8^. All experiments in this study utilized this iPS-RPE line.

THP1-Dual and THP1-Dual KO-cGAS cells were purchased from InvivoGen (San Diego, CA), and cells were prepared and cultured following the manufacturer’s protocols. In short, cells were expanded in suspension cultures in RPMI-1640 supplemented with 10% heat-inactivated fetal calf serum (FBS, Sigma-Aldrich, St. Louis, MO) and 1% penicillin/streptomycin (Gibco BRL, Grand Island, NY) and 100 µg/ml Normocin (InvivoGen, San Diego, CA) at 37 °C and 5% CO2. Cells were passaged every 3 days until seeding into vessels for experimentation.

### Transepithelial resistance (TER) and macrophage-RPE co-culture system

Human RPE cells were seeded (200 k cells/cm^2^) in transmembrane wells (Corning #353,095) and allowed to expand and mature for at least 3 weeks in serum free RtEBM media. The density of RPE post-maturation reached approximately 1800 k cells/cm^2^ or 600 k cells per well for 24 well plate. Barrier function was assessed by monitoring transepithelial resistance (TER) every 15 min by means of a cellZscope 2 (NanoAnalytics GmbH, Münster, Germany). The resistance values for individual monolayers at specific times (Ω/cm^2^) were determined, subtracted for background resistance produced by the blank filter and culture medium (as 0%), and normalized to baseline resistance prior to stimulation (as 100%). For co-culture, human macrophages (300 k cells per well for 24 well plate) were treated and seeded into the basal compartment of a tissue culture plate, or cellZscope2 24-well inserts for resistance measurements.

### Reagents

dsDNA-EC, ISD, VACV-70, G3-YSD, poly(dA:dT) and 3p-hpRNA were purchased from InvivoGen. Nucleic acid was isolated from THP1 cells using MagMAX isolation procedure according to manufacturer’s instructions (AM1840, Thermo Scientific), then treated with RNase I (EN0601, Thermo Scientific). Cells were transfected with indicated nucleic acids using Lipofectamine 3000 (Thermo Fisher Scientific, Waltham, MA). Pro-inflammatory recombinant cytokines were purchased from R&D Systems (Minneapolis, MN): TNFα (#210-TA), IL-1β (#201-LB), IL-18 (#9124-IL), IL-6 (#206-IL), IL-12 (#219-IL), IFNβ (#8499-IF).

### ELISA

Unless otherwise specified, cell lysates and conditioned cell culture supernatants were harvested 18–24 h post transfection. Whole-cell lysates were extracted using cell lysis buffer (Cell Signaling Technology, Danvers, MA; CST#9803), supplemented with protease and phosphatase inhibitors (ThermoFisher Scientific, Waltham, MA) according to the manufacturer’s protocol. All samples were stored at − 80 °C before use. Lysates or supernatant were measured in 384-well plates using antibody pairs for ISG15 (R&D #AF4845, #A-830), and DuoSet kits for human IFNβ (R&D #DY814), TNFα (R&D #DY210), IL1β (R&D #DY201), IL18 (R&D #DY8936-05), IL12 (R&D #DY1270), or IL-6 (R&D #DY206). Protein levels were measured according to manufacturer instructions.

### qPCR

For cultured cells, mRNA was isolated from cells using TurboCapture 96 mRNA Kit (Qiagen, Hilden, Germany; #72,251). For human tissue, RNA was isolated using RNeasy Plus Mini Kit (Qiagen #74,136). cDNA synthesis was performed using High-Capacity cDNA Synthesis Kit (Applied Biosystems, Waltham, MA; #4,368,813). Real-Time PCR was performed using FAM-labeled TaqMan probes targeting genes of interest and a VIC-labeled TaqMan probe targeting β-actin as control (Applied Biosystems). Reactions were run with TaqMan Fast Advanced Master Mix on the ViiA7 system (Applied Biosystems) according to the instructions of the manufacturer.

### ZO-1 staining and imaging analysis

RPE cells grown on membranes were fixed in 4% paraformaldehyde, blocked with 2% BSA (Sigma) for 1 h, and stained overnight at 4 °C with FITC labeled anti-ZO-1 antibody (Thermo Fisher #339111). Membranes were mounted in SlowFade Diamond Antifade Mountant (Thermo Fisher) and imaged using confocal laser-scanning microscopy on a Zeiss Airyscan detector. Roundness and form factor were measured to assess changes in RPE cell morphology with cell boundaries defined by ZO-1 staining. Cells with incomplete boundaries or bordering the edge of the image field were discarded. For roundness, cell shape was analyzed using Arivis Vision4d image processing software. Cell roundness were reported during the segmentation process by Arivis software. RPE form factor was obtained using CellProfiler^19^. Cell area (μm^2^) and cell perimeter (μm) were recorded, and cell density [(# of cells)/(sum of cell areas in μm^2^)], and form factor [(4π * cell area)/(cell perimeter^2^)] were calculated. At least 3 representative images for each biological triplicate were analyzed for each condition, and each experiment was performed at least 5 times. One representative independent experiment is presented in the manuscript.

### Data analysis

Protein levels measured by ELISA were presented as absolute amount. For TER experiments, three independent experiments with triplicates within each experiment were performed, and values were presented as bar graph with mean ± S.D. Imaging analysis for ZO1 staining pictures were represented as violin plot of each measured cell to detail the morphological changes as a distribution. Two groups were compared using Student's t-test. Multiple comparisons were made using One-Way ANOVA followed by a post hoc *Newman–Keuls* test. Differences were considered significant at *p* < 0.05.

### Ethical approval

All procedures performed in studies involving human participants were in accordance with the ethical standards of the institutional and/or national research committee.

### Informed consent

Postmortem human eyes were procured by the Lions Eye Institute for Transplant & Research (Tampa, FL, USA) with consent of donors or donors’ next of kin and in accordance with the Eye Bank Association of America medical standards, US/Florida law for human tissue donation, the Declaration of Helsinki and Food and Drug Administration regulations, and Novartis human tissue registration working practice guidelines regarding research using human tissues.

Human blood was obtained from Medcor Cambridge Research Donor Program using the Western Institutional Review Board (WIRB) Protocol #2,015,286 and WIRB-approved patient informed consent. The WIRB protocol complies with all federal, state, and local laws pertaining to human research.

### Supplementary Information

Supplementary Figures.

## Data Availability

The datasets used and/or analyzed during the current study are available from the corresponding author on reasonable request.
