# Supplementary material for: TNFα induced by DNA-sensing in macrophage compromises retinal pigment epithelial (RPE) barrier function
Source: Sci Rep. 2023 Sep 2;13:14451. doi: 10.1038/s41598-023-41610-7 (PMC10475136; doi:10.1038/s41598-023-41610-7)
Supplement: Supplementary file 1 — Supplementary Figures. [file 41598_2023_41610_MOESM1_ESM.pdf]

TNFα induced by DNA-sensing in macrophage compromises retinal pigment epithelial (RPE) barrier function

Michael Twarog, Joshua Schustak, Yongyao Xu, Matthew Coble, Katie Dolan, Robert Esterberg, Qian Huang, Magali Saint-Geniez, Yi Bao*

From the Department of Ophthalmology, Novartis Institutes for BioMedical Research, 22 Windsor Street, Cambridge, MA, U.S.A.

* Corresponding author: Yi Bao, email: yi.bao@novartis.com

Supplementary information

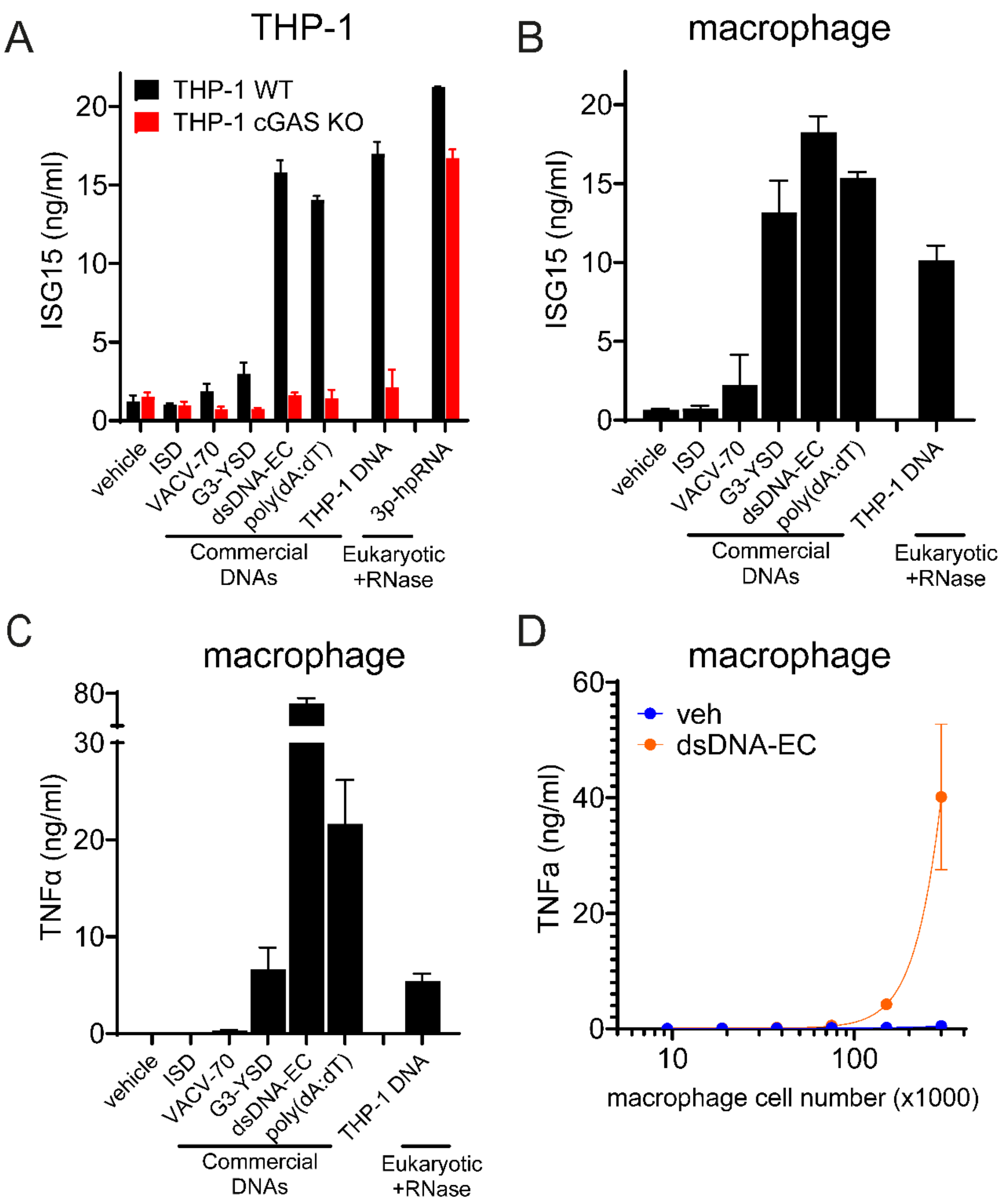

Supplementary Figure 1. Optimization of cGAS activation and macrophage numbers for RPE-macrophage co-culture system. (A-C) Different source of immunogenic DNAs (200 ng/ml for commercial DNAs and 28 ng/ml for DNA isolated from THP1) were evaluated on both THP1 cells (A) and human primary macrophages (B-C) for IFN (A-B) and TNF (C) responses. (D) 300 k macrophages were required to generate sufficient TNFα (> ng/ml) in the medium in response to DNA challenge.

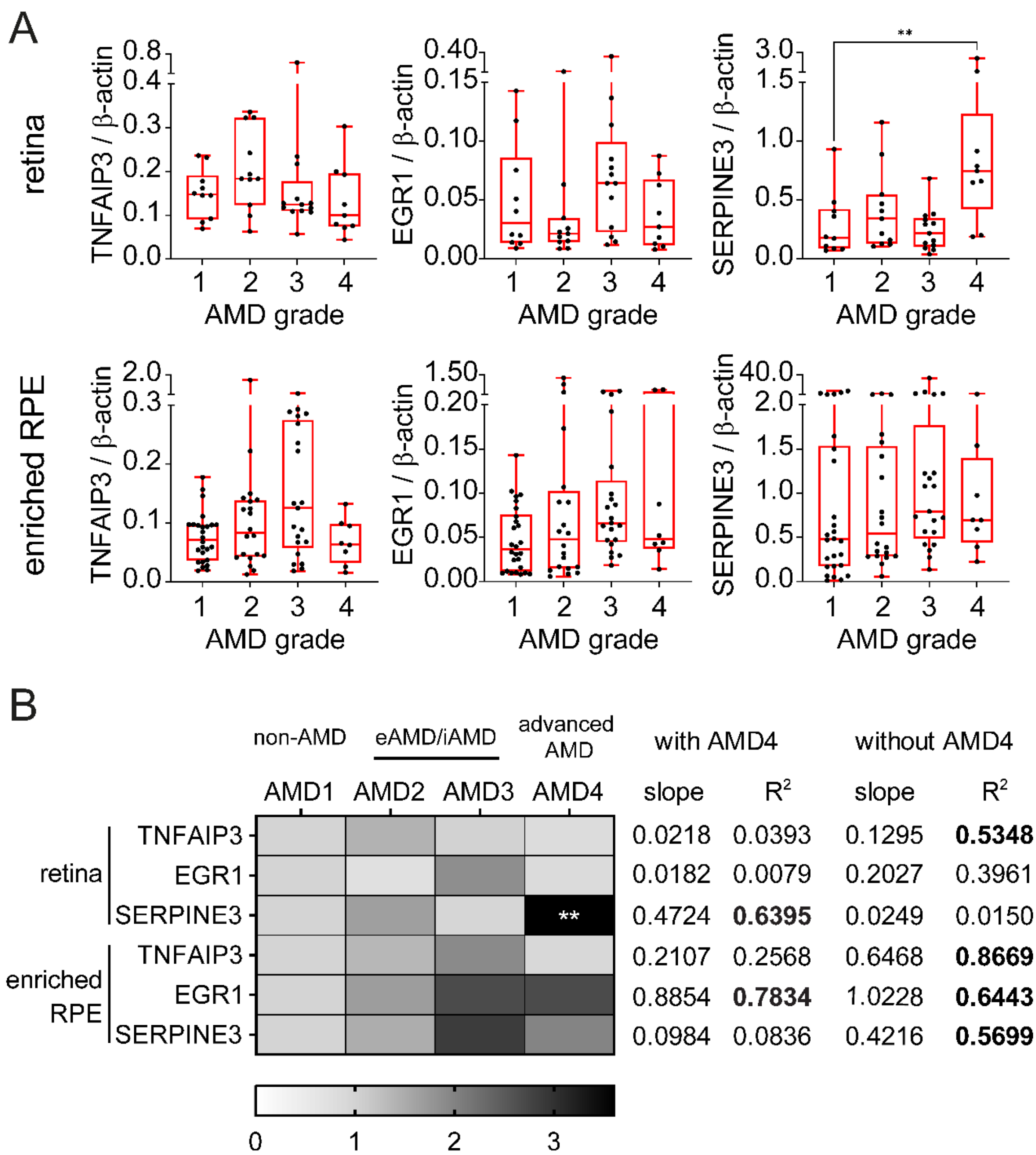

		with AMD4 slope	with AMD4 R^2	without AMD4 slope	without AMD4 R^2
retina	TNFAIP3	0.0218	0.0393	0.1295	**0.5348**
retina	EGR1	0.0182	0.0079	0.2027	0.3961
retina	SERPINE3	0.4724	**0.6395**	0.0249	0.0150
enriched RPE	TNFAIP3	0.2107	0.2568	0.6468	**0.8669**
enriched RPE	EGR1	0.8854	**0.7834**	1.0228	**0.6443**
enriched RPE	SERPINE3	0.0984	0.0836	0.4216	**0.5699**

Supplementary Figure 2. TNFα signature genes trend up in patients with increasing AMD grades. (A-B) Expression of TNF-associated genes were evaluated by qPCR in the retina and enriched RPE samples from AMD patients grades 1 to 4.(A) Distributions are presented as box-and-whisker plots of individual patient samples (n = 9-13). **, *p*<0.01. (B) Summary heat-map using the geometric mean value of all data points indicated in relative to AMD1. To better understand the trend of gene changes, we conducted a linear regression analysis to assess correlations. We calculated both the slope and R^2 values for the data, with and without AMD4 group. We highlighted data with an R^2 value >0.5 to demonstrate a stronger trend of upregulation in samples from AMD patients, particularly in enriched RPE. AMD1 – non-AMD patients; AMD2/3 – early or intermediate stage of AMD (eAMD/iAMD); AMD4 – advanced AMD.
